# Salivary microRNA miR-let-7a-5p and miR-3928 could be used as potential diagnostic bio-markers for head and neck squamous cell carcinoma

**DOI:** 10.1371/journal.pone.0221779

**Published:** 2020-03-24

**Authors:** Rushdi S. Fadhil, Ming Q. Wei, Dimitrios Nikolarakos, David Good, Raj G. Nair

**Affiliations:** 1 School of Medical Science, Griffith University and Menzies Health Institute Queensland, Gold Coast, Queensland, Australia; 2 School of Dentistry and Oral Health, Griffith University Gold Coast, Queensland, Australia; 3 Oral and Maxillofacial Surgery, Queensland Health, Gold Coast University Hospital, Gold Coast, Queensland, Australia; 4 Discipline of Physiotherapy, School of Allied Health, Australian Catholic University, Queensland, Australia; 5 Oral Oncology, Haematology and Oncology, Gold Coast University Hospital, Gold Coast, Queensland, Australia; University of South Alabama Mitchell Cancer Institute, UNITED STATES

## Abstract

**Backgrounds:**

MicroRNAs (miRNA) are a class of non-protein-coding RNAs that have significant biological and pathological functions. The importance of miRNAs as potential cancer diagnostic biomarkers is gaining attention due to their influence in the regulation of cellular processes such as cell differentiation, proliferation and apoptosis. The aim of this study was to identify significant miRNAs from saliva as potential diagnostic biomarkers in the early diagnosis and prognosis of head and neck squamous cell carcinoma (HNSCC).

**Materials and methods:**

Five differentially expressed miRNAs (miR-7703, miR- let-7a-5p, miR- 345-5p, miR- 3928 and miR- 1470) were selected from Next Generation Sequencing (NGS) miRNA data generated from our previous study using saliva of 12 HNSCC patients and 12 healthy controls. Their differential expressed miRNAs were subsequently validated by RT-qPCR using saliva samples from healthy controls (n = 80) and HNSCC patients (n = 150). Total RNA was isolated from 150 saliva samples of HNSCC patients and was transcripted into cDNA by TaqMan MicroRNA Reverse Transcription Kit. Using quantitative RT-PCR analysis, salivary miRNAs were identified in HNSCC patients (n = 150) and healthy controlled cases (n = 80). T-tests were used to compare the differences among the various clinical variants.

**Results:**

On average 160 ng/μl was isolated from 500 μl of saliva. Overall, a good correlation observed between the HNSCC and some of miRNAs expression levels. Salivary miR-let-7a-5p (P<0.0001) and miR-3928 (P< 0.01) were significantly down regulated in saliva of HNSCC patients relative to age and sex-matched healthy controls. A number of salivary miRNAs (miR-let-7a-5p and miR-3928) were correlated with lymph node metastasis (p = 0.003, p = 0.049) and tumour size (p = 0.01, p = 0.02), respectively. However, our preliminary analysis showed no significant differences in salivary miR-1470, miR-345-5p or miR-7703 expression between patients and healthy controls. Most notably, our analysis showed that salivary miR-let-7a-5p and miR-3928 expression levels have significant sensitivity and specificity to distinguish between patients with HNSCC and healthy controls.

**Conclusion:**

This study concluded that salivary miR-let-7a-5p and miR-3928 has the potential to be novel non-invasive biomarkers for early detection and prognosis of HNSCC.

## Introduction

Head and neck squamous cell carcinoma (HNSCC) is the fifth most frequent carcinoma in men worldwide and includes tumours at various sites in the oral and upper aerodigestive tract[1] with annual reported deaths of about 300,000 worldwide[2, 3]. The asymptomatic nature of the tumour leads to late diagnosis, extensive lesions and potential metastasis even though there may well be early salient potentially malignant disorders preceding [4]. Five year survival remains a challenge with compromised prognosis and deaths [5]. Improved prognosis of HNSCC requires early diagnosis through clinical examination and biomarker discovery [6].

Salivary biomarkers are ideal non-invasive diagnostic tool for diseases including cancer [4, 7–10]. The ease of collection and its non-invasive nature making salivary biomarkers a cheap and reliable alternative when compared with traditional samples such as blood and tissue for diagnosis and prognosis [11]. Furthermore, it is an ideal tool for large scale sample collection, epidemiological screening and is relatively economical when it comes to long term monitoring [12]. It has been reported that more than 3,000 species of RNA are expressed in saliva majority of which are microRNAs (mRNAs) [13], and recent reports support its role in oral cancer progression and various cancers [14, 15, 16].

Since its discovery in 1993, miRNAs have been considered a novel regulator biomarker for gene expression [17]. MiRNA is a small non-protein-coding RNA containing 19–24 nucleotides that control the degradation and translation of RNA [18]. Evidence indicated that miRNA has an important role in carcinogenesis [19, 20]. It has also been predicted that more than one thousand miRNAs may be found in the human genome[20, 21]. Although multiple mRNAs could be regulated by a single miRNA, few biological pathways are not influenced by miRNAs. The aberrations in miRNA expression and their apparent pluripotent roles predict that miRNA identification in oncogenesis might reveal networks or targets that are suitable for cancer diagnostic markers or cancer therapy [20, 22]. In addition, it has been stated that there is a unique miRNA biomarker for each cancer type [23]. For instance, correlation has previously been found between vascular invasion of HNSCC and miR-211 [24]. Moreover, miR-184 and miR-21 have been identified to be oncogenic miRNAs in HNSCC [25]. From our initial study using next generation sequencing (Qiagen), a subset of five differentially expressed miRNAs (miR-7703, miR- let-7a-5p, miR- 345-5p, miR- 3928 and miR- 1470) have been selected (based on significant differential fold change) to determine their clinical utility as a potential biomarker.

miR-let-7a-5p was found up-regulated in human prostate cancers and this miRNA accompanied the prostate oncogenic process [1]. However, a recent study demonstrated the down-regulation of miR-let-7a-5p in a wide variety of neoplasms including colorectal carcinoma (CRC), breast cancer and lung cancer [26, 27]. miR-3928 has been demonstrated to be induced by ionizing radiations in HeLa cells and targeted the endoribonuclease Dicer [28]. miR-345-5p was shown to be expressed at substantially lower levels among the 34 predicted cytidine deaminase (CDA) associated miRNAs [29].

## Materials and methods

### Participants

Unstimulated whole saliva was collected before surgery from patients attended the Gold Coast University Hospital (GCUH) in Gold Coast, Queensland with HNSCC (n = 150) at different stages of the disease, from different sites (glottis, buccal sulcus, buccal mucosa, tongue, and floor of the mouth (FOM). Similarly, whole saliva samples were collected from healthy controls (n = 80) without a history of any malignancy, and who had good oral hygiene. In addition to verbal, written informed consent obtained from all participants. Samples collected from a period of December 1, 2015, to May 30, 2018, from the GCUH. HNSCC were classified according to the extent or size of tumour (T), spread to the regional lymph nodes (N) and the presence or absence of metastasis (M) according to TNM classification (American Joint Committee on Cancer) [30]. The clinical demographic data of all participants are listed in Table 1. This study was approved by HREC of Griffith University (GU Ref No: 2015/766) and Gold Coast University Hospital (HREC/15/QGC/223), Queensland, Australia.

**Table 1:** Demographic and clinical characteristics of HNSCC patients.

Characteristic	HNSCC 150 (%)	Control 80 (%)	P value
**Sex**			
Male	90 (60)	35 (43.7)	0.1
Female	60 (40)	45 (56.2)	
**Age**			
≥ 50	95 (63.3)	42 (52.5)	0.2
< 50	55 (36.6)	38 (47.5)	
**Smoking history**			
Current	90 (60)	29 (36.2)	0.34
Former	45 (30)	25 (31.2)	
Never	15 (10)	26 (32.5)	
**Alcohol consumption**			
Non-drinker	60 (40)	20 (25)	0.09
Former drinker	60 (40)	35 (43.7)	
Current drinker	30 (20)	25 (31.25)	
**Alcoholic & Smoking**			
Yes	35 (23.3)	10 (12.5)	0.5
No	115 (76.6)	70 (87.5)	
**Tumour Stage**			
I	49 (32.6)		
II	13 (8.6)		
III	0 (0)		
IV	88 (58.6)		
**N status**			
N0	88 (58.6)		
N+	62 (41.3)		
**HPV status**			
HPV16 +ve	35 (23.3)		
HPV16 -ve	25 (16.6)		
HPV status missing	90 (60)		
**Total**	**150 (100)**		

### Saliva collection

Whole saliva from participants were collected in 50-mL sterile falcon tube between 6 am to 11 am, as previously described [31, 32]. Participants were asked to refrain from eating, drinking, any oral hygiene procedures. Five minutes prior to sample collection, participants were asked to swish their mouth gently with sterile water, provided, to minimize contamination five minutes before sample collection. Participants were seated upright comfortably on a chair and asked to spit into a 50-mL RNA/DNA free Falcon tube kept on a tray with ice. A maximum of 8 mL of whole saliva was collected from each subject within a duration of 20–30 min. Samples were immediately stored in dry ice and sent for processing in the laboratory.

### Salivary RNA extraction

RNeasy kit (Qiagen, Valencia, CA, USA) was used according to the manufacturer’s protocol to extract total RNA from the collected saliva for each subject (440μl). Spectrophotometer (NanoDrop, ND2000; Wilmington, DE, USA) was used to validate the purification and quantification of the isolated RNA.

### Reverse transcription

Complementary DNA was generated *via* a miRNA reverse transcription kit (Origene, HP) using total RNA (10–50 ng). The10-μl RT reaction mixture contained 1 to 2μg of total RNA, 1 μl of poly A tailing buffer, 1 μl of mM ATP, 1 μl of Poly (A) polymerase and adjusted by molecular water. The polyadenylation reaction continued at 37°C for 2 hours. 1μl of oligo dT primer was then added to the tube, incubated at 70°C for 5 minutes and placed in dry ice for 2 minutes. For final cDNA synthesis 4 μl of 5X MMLV buffer was added, then incubation at 42°C for 1 hour, 95° for 5 minutes and then placed in dry ice. The reaction mixture was diluted with 200μl nuclease- free water and kept at -20°C for real-time quantitative polymerase chain reaction (RT-qPCR).

### Real-time quantitative polymerase chain reaction

To evaluate miRNA, candidates from previous studies RT-qPCR, were analysed. RT-qPCR processing of the miRNAs used specific forward and universal reverse primers based on the manufacture's protocol (Origene). All primers used in this study are mentioned in Table 2. PCR amplicons were investigated by SYBR Green using the level of fluorescence emitted. Reactions were performed in triplicate with cycling conditions as follows: 95°C for 30 seconds then 95°C for 15 seconds, 55°C for 10 seconds, and 72°C for 30 seconds, repeated 42 times. The melt curve analysis was set to validate the expected generation of the PCR product. The setting was 60.0 to 95.0°C at increments of 0.5°C for 0.05 minutes. The Ct values were < 35. A non-template control (NTC) was used as a negative control. Quant Studio 6 flex (Applied Biosystems) was used to carry out the RT-qPCR reactions and Δct was calculated and normalized using housekeeping miRNA-16.

**Table 2:** Sequence, genomic location and primers of novel miRNAs.

miRNA	Mature sequence 5′–3′	Forward primer	Reverse Primer
miR-7703	UUGCACUCUGGCCUUCUCCCAGG	TTGCACTCTGGCCTTCTCC	GAACATGTCTGCGTATCTC
miR-7a-5p	UGAGGUAGUAGGUUGUAUAGUU	GCAGTGAGGTAGTAGGTTGT	GGTCCAGTTTTTTTTTTTTTTTAACTATAC
miR-3928	UGAAGCUCUAAGGUUCCGCCUGC	TGAAGCTCTAAGGTTCCGCC	GAACATGTCTGCGTATCTC
miR-1470	GCCCUCCGCCCGUGCACCCCG	CCTCCGCCCGTGCAC	GAACATGTCTGCGTATCTC
miR-345-5p	GCUGACUCCUAGUCCAGGGCUC	CGCAGTACAGTACTGTGATAAC	AGGTCCAGTTTTTTTTTTTTTTTCAG
miRNA-16	UAGCAGCACGUAAAUAUUGGCG	AGCAGCACGTAAATATTGG	GAACATGTCTGCGTATCTC

### Statistical analyses

All data analysis was performed using Prism software, version 6. Samples were treated as normal and progressive HNSCC. Data normalized threshold cycle number ΔCt, where ΔCt = [Ct (Target miRNA)]- [Ct (miR-16)], and the relative miRNA expression level were calculated as 2^-(ΔCt) which is commonly used in miRNAs detection studies. The p-value less than 0.05 was considered as significant.

## Results

### Characteristics of study subjects

In total, there were 150 saliva samples from HNSCC patients admitted to the GCUH and 80 saliva samples were collected from healthy matching controls, students and staff of Griffith University. The tumour localizations, histopathological features, treatment options, and demographic properties of the HNSCC patients are listed in Table 1. The mean age of the HNSCC patients was 60.5 ± SD years, while the mean age of the healthy controls was 56.6 ± SD years where P = 0.2 (Table 1). The HNSCC group consisted of 90 men and 60 women, and the healthy controls consisted of 35 men and 45 women. In addition, there were no significant differences between these groups in terms of smoking and alcohol consumption (p = 0.09) habits.

### The expression of the five candidate miRNAs from HNSCC patients and healthy controls

**Fig 1:**
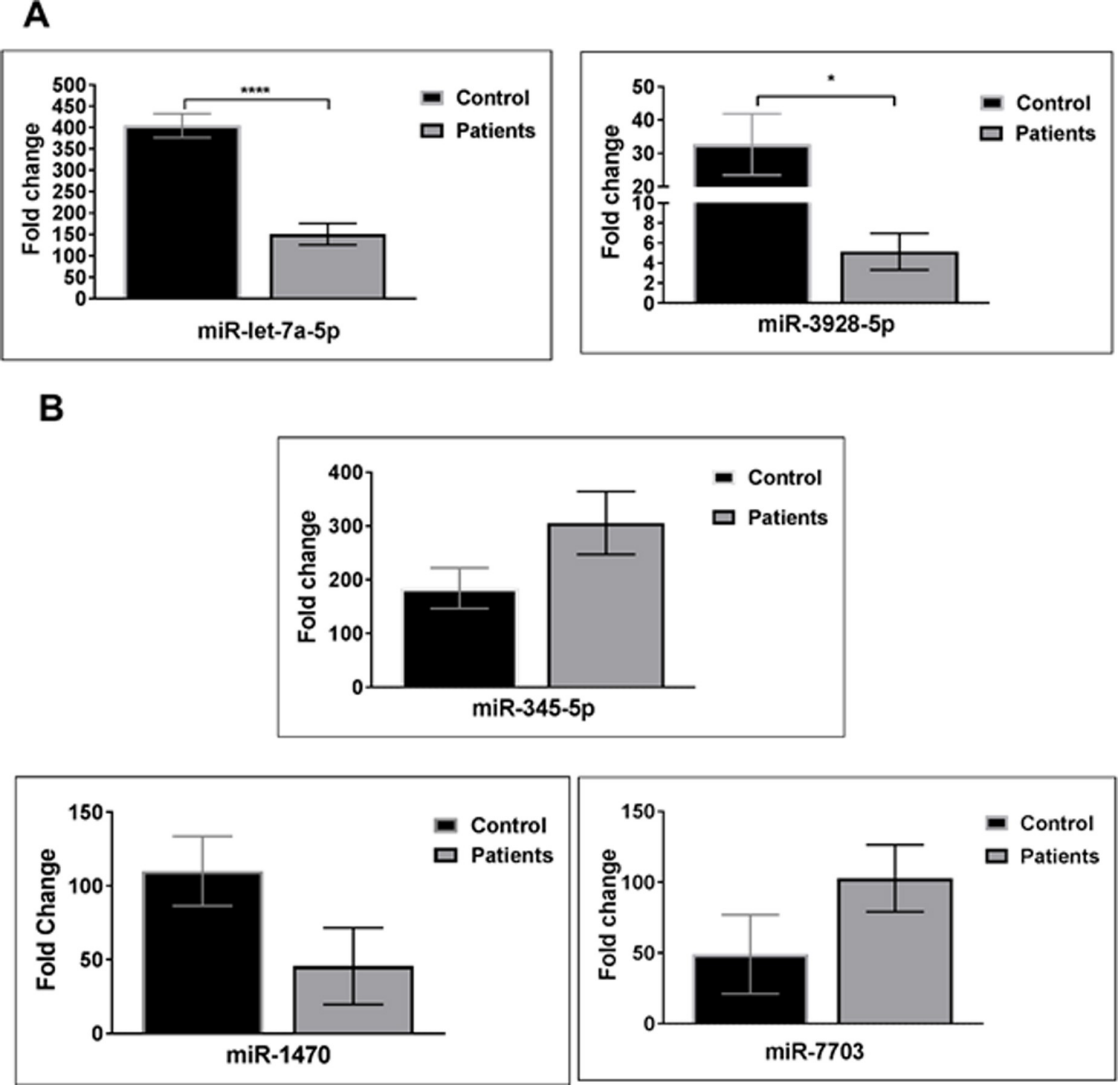
Expression of candidate miRNA in supernatant saliva of subjects (n = 150) vs. control (n = 80). (A) Significant decrease of miR-let-7a-5p, miR-3928. (B) Expression of miR-345-5p, miR-1470 and miR-7703, p-values is indicated as follows: * p < 0.05 and **** p < 0.001 (t test).

Based on previous studies performed on profiling miRNAs from different samples and their potential as targets and functions on oral cancer, five salivary miRNAs (miR-7703, miR- let-7a-5p, miR- 345-5p, miR- 3928 and miR- 1470) were selected for further confirmation using the RT-qPCR technique. We investigated these five validated neoplasm-associated miRNAs in supernatant saliva from both patients with HNSCC and controls groups. Two validated miRNAs (miR- let-7a-5p, miR- 3928) were significantly down regulated (Fig 1). Moreover, the other three validated miRNAs (miR-7703, miR-345-5p and miR-1470) were shown to be slightly dysregulated (up or down), although not significant in the HNSCC group compared with the healthy controls where p value were 0.13, 0.07, 0.09, respectively (Fig 1).

### Discriminatory power of miR-let-7a-5p and miR-3928

**Fig 2:**
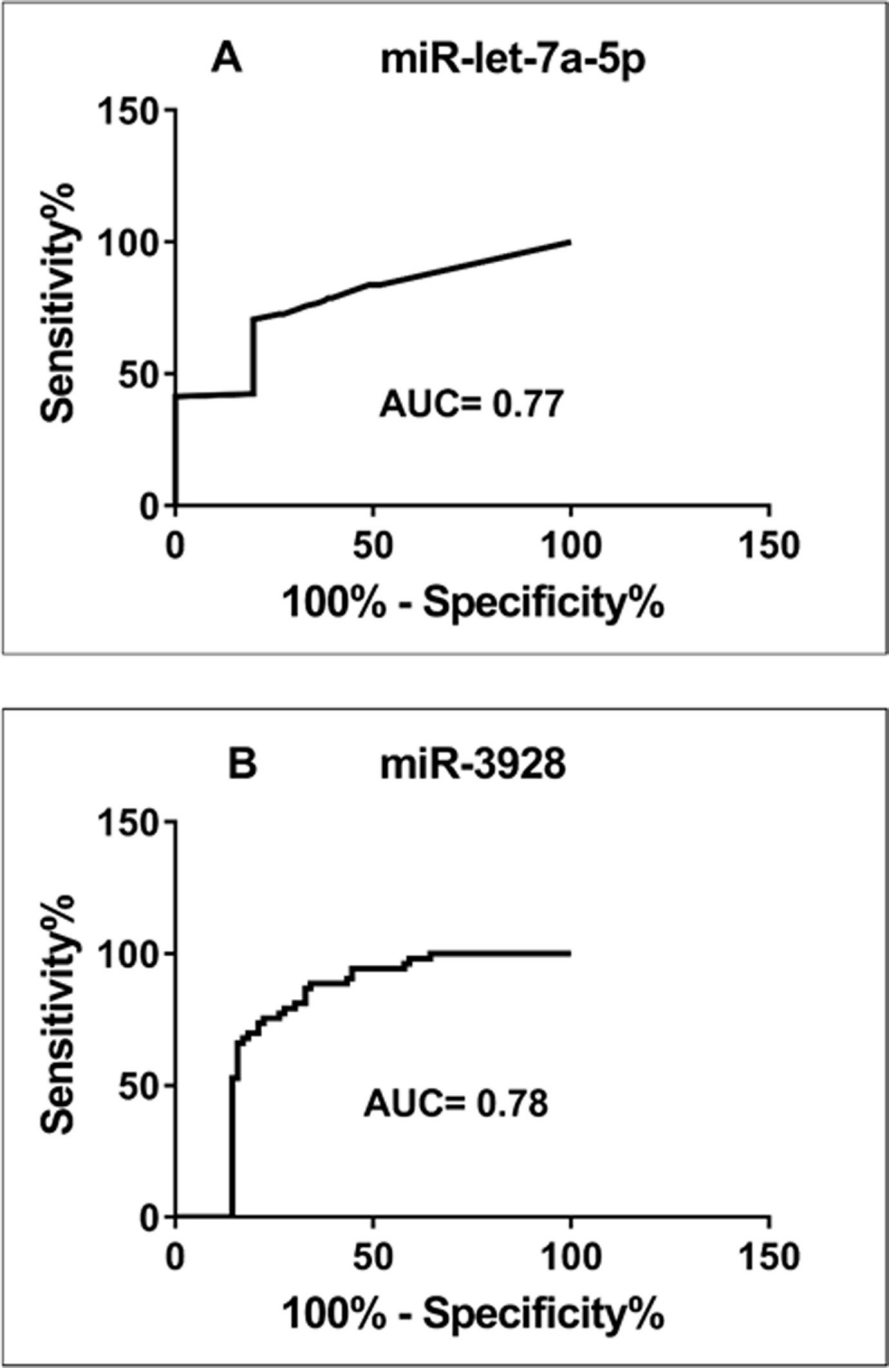
Receiver operator curve (ROC) analysis of the independent validation study using saliva-derived (A) miR-let-7a-5p and (B) miR-3928.

**Fig 3:**
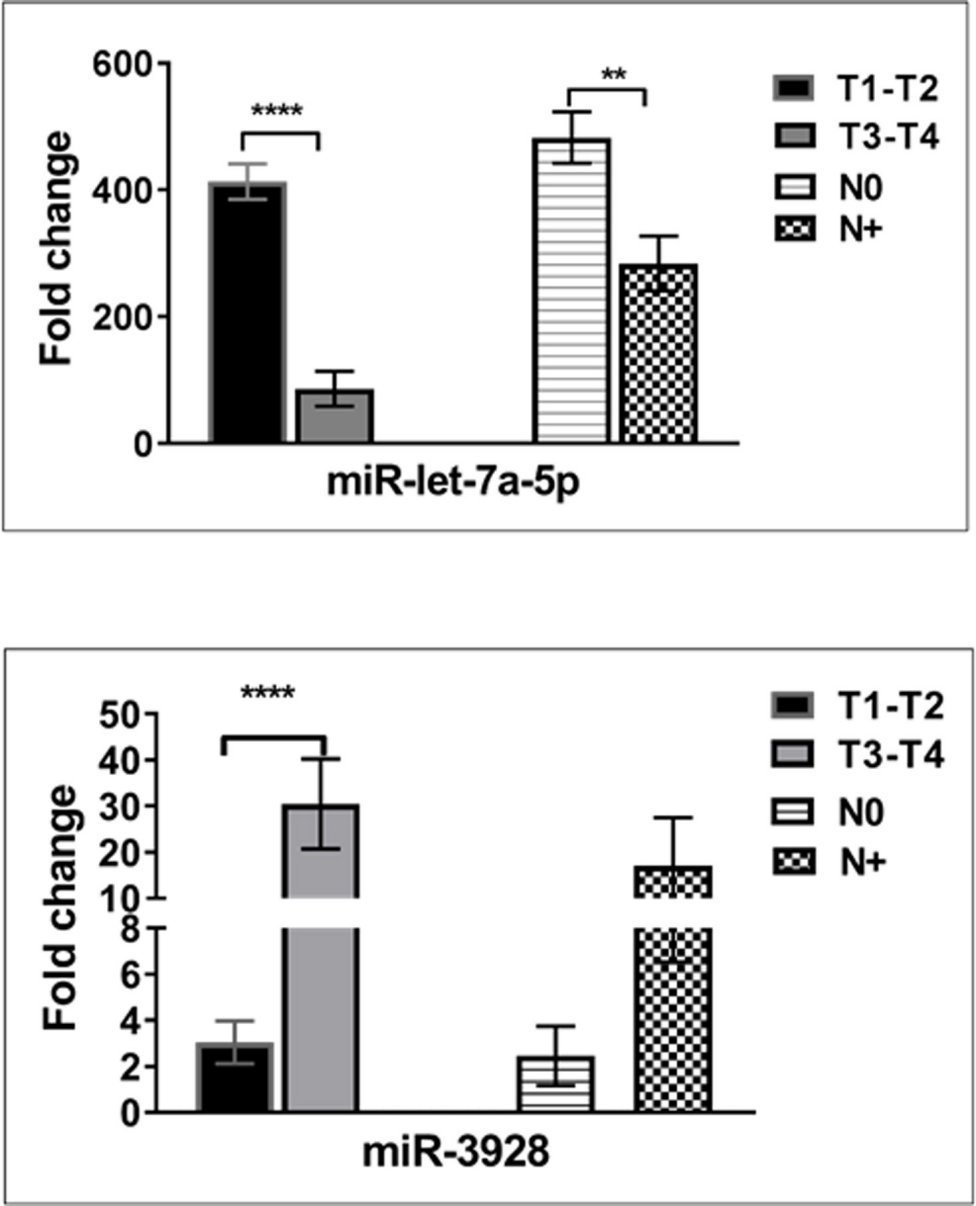
Expression of miR-let-7a-5p and miR-3928 in different groups of HNC patients (n = 150). HNC patients were categorized according to clinical stage (T) and the presence [N+] and absence [N0] of lymph node metastasis. Significance of two-sided p-values indicated as follows: ** p < 0.01, **** P<0.0001 (t test).

**Fig 4:**
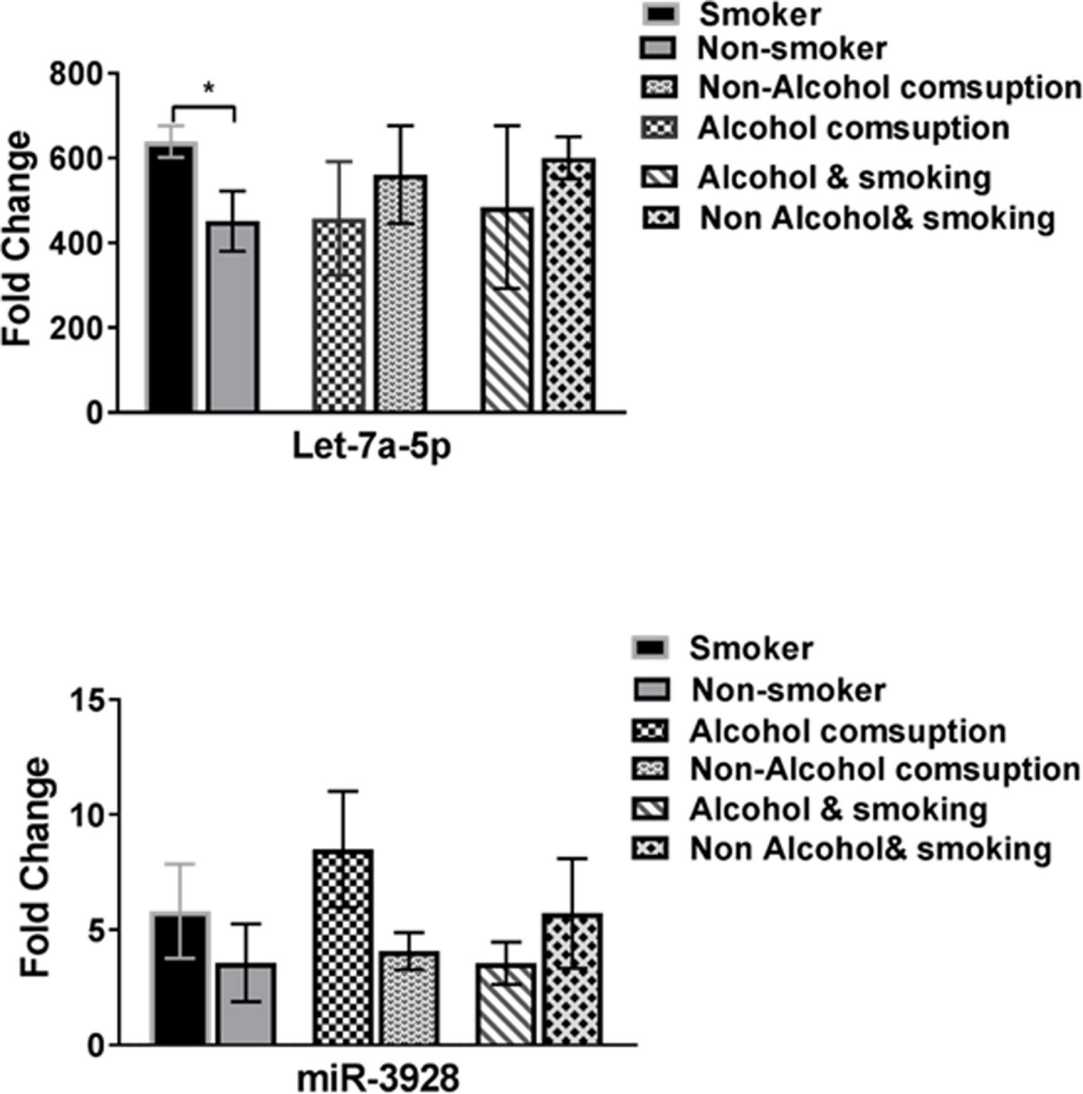
Expression of miR-let-7a-5p and miR-3928 in different groups of patients (n = 150) were classified by the habit of smoking and alcohol consumption. Significance of two-sided p-values is indicated as follows: * p < 0.05 (t test).

Receiver Operator Curves (ROC) were created to evaluate the discriminatory power of miR- let-7a-5p and miR- 3928 for their ability to distinguish between control and disease groups. These miRNAs were revealed to provide a good discriminative ability with AUC values of 0.85and 0.74, respectively. Fig 2 shows ROC analyses for these two miRNAs using supernatant salivary samples from all 150 HNSCC patients and 80 healthy controls. Interestingly, this study has revealed that salivary miR-let-7a-5p was significantly influenced by cancer staging and lymph node metastasis (Fig 3). Furthermore, a closer look at the miR-3928 in this study showed that, in the late stage tumor group, the down-regulation of the miRNA had a significant decrease in expression compared with first tumor stage model of patients with HNSCC while no significant differences has been revealed in metastasis model of this miRNA (Fig 3). Expression of miR- let-7a-5p and miR- 3928 on epigenetic variables such as smoking and alcohol consumption when compared was statistically different (p = 0.05; Table 1 and Fig 4).

## Discussion

There are no saliva-based biomarkers available in the early detection or as a prognostic indicator for HNSCC. We are reporting two novel biomarkers (miR- let-7a-5p and miR- 3928) in saliva that we had identified to be a reliable with a potential to aid in the diagnosis and prognosis of HNSCC. This adds credibility to the existing knowledge on low level expression of miR-let-7a and miR-3928 as well as the identified list of targeted oncogenes or suppressor genes [33, 34]. This, to our knowledge, is the first report on these two salivary miRNAs in patients with HNSCC. Another study mentioned that miR-let-7a down regulates the miR-21 expression [35], an oncogenic miRNA that was highly expressed in different types of cancer[36, 37]. Interestingly, miR-let-7a was also the most downregulated miRNA amongst others in plasma sample of patients with colorectal cancer when compared with normal controls using qRT-PCR [38]. In addition, it has been reported by strong evidence that miR-let-7a targeted genes in several types of cancer, for instance, CASP8, BCL2, CCND2, EWSR1, FOXA1, DICER1, HMGA1, HMGA2, KRAS, MYC, HRAS, MPL, NF2, PRDM1, NRAS [39]34 and COSMIC [40]. It is known that, different malignancies are initiated by dysregulation of the p53 tumour suppressor protein. Many genes are altered in expression by p53 deregulation which lead to cell-cycle arrest, inhibition of angiogenesis and apoptosis [41]. Zhang and colleagues have reported that p53 regulates different genes such as miR-let-7a, miR-34a, caspase3 and bcl2 [42].

However, cell proliferation has been inhibited by the increased level of miR-let-7a in the cell line of pancreatic cancer [43]. Several studies have reported that let-7a could inhibit the growth of cells in lung cancer and lymphoma [44, 45] and STAT3 signalling was influenced by miR-let-7a in cervical carcinogenesis.[46]. Transfection of let-7a-1 precursor miRNA lead to inhibited expression of c-myc and RAS proteins and cause of significant suppression in growth. [47].

We report significant down-regulation of miR-3928 in the supernatant saliva samples from HNSCC patients. Tumour inhibition role of miR-3928 has been demonstrated in osteosarcoma [33]. It has been reported that the expression of miR-3928 was down-regulated, and high level of miR-3928 inhibited the growth of tumour in osteosarcoma tissue [33]. Another study has reported that activation of phosphorylated checkpoint kinase 1 (Chk1), Rad3-related kinase (ATR) and DNA damage are induced by upregulation of miR-3928 accompanied by G1 arrest. [28].

Recent reports confirmed that, HBX induces miR-3928v through NF-κB/EGR1 signalling pathway and accelerated hepatocellular carcinoma by decreasing the expression of suppressor gene VDAC3 using calorimetry, colony formation, migration/invasion, vascular mimicry assays *in vitro* and xenograft tumour model *in vivo* using hepatocellular carcinoma cell lines [48]

## Conclusion

Our results concluded that the expression levels of the miR-let-7a-5p and miR-3928 were significantly decreased in supernatant saliva samples of patients with HNSCC compared with the healthy matched controls. Furthermore, ROC curve analysis revealed that slivery miR-let-7a-5p and miR-3928 expression levels have significant sensitivity and specificity to differentiate between HNSCC patients and healthy controls. This study suggests that the downregulation of miR-let-7a-5p and miR-3928 in patients with HNSCC could be useful tool as an early diagnostic aid and prognostic indicator for HNSCC. Further studies using a larger number of patients, different sites and stages of HNSCC are necessary before elucidating the full potential of the above two miRNAs as salivary biomarkers for HNSCC.
